# How much BiGAN and CycleGAN-learned hidden features are effective for COVID-19 detection from CT images? A comparative study

**DOI:** 10.1007/s11227-022-04775-y

**Published:** 2022-08-26

**Authors:** Sima Sarv Ahrabi, Alireza Momenzadeh, Enzo Baccarelli, Michele Scarpiniti, Lorenzo Piazzo

**Affiliations:** Department of Information Engineering, Electronics and Telecommunications, Sapienza University or Rome, Via Eudossiana, 18, 00184 Roma, Italy

**Keywords:** COVID-19 detection, Hidden feature extraction, BiGAN, CycleGAN, CAE, Unsupervised-vs.-weakly supervised learning, Complexity-vs.-accuracy comparisons

## Abstract

Bidirectional generative adversarial networks (BiGANs) and cycle generative adversarial networks (CycleGANs) are two emerging machine learning models that, up to now, have been used as generative models, i.e., to generate output data sampled from a target probability distribution. However, these models are also equipped with encoding modules, which, after weakly supervised training, could be, in principle, exploited for the extraction of hidden features from the input data. At the present time, how these extracted features could be effectively exploited for classification tasks is still an unexplored field. Hence, motivated by this consideration, in this paper, we develop and numerically test the performance of a novel inference engine that relies on the exploitation of BiGAN and CycleGAN-learned hidden features for the detection of COVID-19 disease from other lung diseases in computer tomography (CT) scans. In this respect, the main contributions of the paper are twofold. First, we develop a kernel density estimation (KDE)-based inference method, which, in the training phase, leverages the hidden features extracted by BiGANs and CycleGANs for estimating the (*a priori* unknown) probability density function (PDF) of the CT scans of COVID-19 patients and, then, in the inference phase, uses it as a target COVID-PDF for the detection of COVID diseases. As a second major contribution, we numerically evaluate and compare the classification accuracies of the implemented BiGAN and CycleGAN models against the ones of some state-of-the-art methods, which rely on the unsupervised training of convolutional autoencoders (CAEs) for attaining feature extraction. The performance comparisons are carried out by considering a spectrum of different training loss functions and distance metrics. The obtained classification accuracies of the proposed CycleGAN-based (resp., BiGAN-based) models outperform the corresponding ones of the considered benchmark CAE-based models of about 16% (resp., 14%).

## Introduction

The chest computed tomography (CT) scan is generally regarded as beneficial in diagnosing COVID-19 diseases and is especially useful when it is used in tandem with clinical examinations [1–5]. Due to the effective use of deep learning (DL) in computer vision and biomedical domains, researchers have explored the efficiency of DL-based methods to recognize COVID-19 from lung CT scans. The current DL approaches can be categorized as supervised, unsupervised, or weakly supervised methods.

### Supervised learning approaches

A large number of research papers adopt supervised learning methods for the reliable detection of COVID-19 diseases [6–16] [17–20]. However, due to the lack of publicly available CTs on COVID-19 patients, researchers have been triggered to consider this deficiency, especially at the beginning of the spread of COVID-19. For instance, the authors of [21–30] adopt *transfer learning* methods to address the lack of large-sized data sets. In [31], the authors utilize GoogleNet and ResNet for supervised COVID-19 classification. The authors of [32] propose a statistical method to address issues, like as huge computational complexity and large datasets required by deep networks. In [33], a segmented CT scan is used as the input of a random forest classifier approach. The authors of [21] used an inception network on CT scans, but the resulting classification accuracy was below average. In [34], the authors propose a contrast enhancement scheme for CT scans, followed by a pre-trained VGG16 and AlexNet classification, reporting good accuracy.

However, the accuracy performance of supervised-trained models typically crashes when CT scans are used in the test phase that belong to unseen classes (that is, classes of test data which are not present in the training data sets). In principle, this loss of robustness suffered by supervised DL models may be effectively by-passed by resorting to unsupervised or weakly supervised DL models, which are trained only on data sets of the COVID-19 class. So doing, it expected that an unsupervised/weakly supervised trained model may differentiate the COVID-19 class (i.e., the target class) from any other type of unseen chest images (i.e., the novelties) in a reliable way.

### Unsupervised learning approaches

Autoencoders (AEs) have been employed in [35–40]. Specifically, the work in [37] focuses on a two-stage learning method and a triple classification task. The authors train their AE model on classes of COVID-19, pneumonia, and normal cases separately. After obtaining the hidden feature vectors of all classes, a feature classifier is trained. The authors of [38] build up a robust statistical target histogram by exploiting the feature representations, which are generated by an unsupervised-trained denoising convolutional AE (DCAE). The proposed method estimates the statistical distance between unknown and target histograms to classify the images according to suitably set decision thresholds. The DCAE proposed in [36] is trained on COVID-19, pneumonia, and a few other types of chest X-rays. Then, the hidden feature vector of a test image is compared to the features of the selected training data sets. The so-trained AE exhibits good test performance. However, unlike our work, this approach relies on training the considered model over each decision class and, then, does not guarantee to instances of unseen class.

#### Generative Adversarial Networks (GANs)-based approaches

Motivated by the aforementioned considerations, we are interested in deep generative models, because learning COVID-19 patterns can be viewed as learning the distribution of the available training data. According to a recent taxonomy in medical image classification [41], we adopt the weakly supervised terminology for indicating the exploitation of two sets of unpaired images. Being unsupervised/weakly supervised models, deep generative models (DGMs) aim to unveil meaningful patterns in raw data. DGMs enable the approximation of statistical data distributions through density estimation. Deep neural networks (DNNs) are based only on point estimates and make deterministic predictions by using suitable feature vectors. Most works on DNNs do not pay much attention to the complexity of these models. On the other hand, probabilistic models typically rely on statistical hypothesis tests, which are more simple to implement through the computation of suitable distances in the latent space [42]. The actual capability of GANs to generate data makes them attractive for anomaly detection under two perspectives [43]. First, GANs can potentially help to generate hard-to-acquire anomalous data points. Second, they can be used to learn the distribution of data for normal operating conditions and, then, can be exploited as anomaly or outlier detectors [44]. A conditional GAN-based model, called CovidGAN, is proposed in [45], which generates synthetic chest X-ray images to augment the available training set. The authors of [46] develop a Dense GAN and a multi-layer attention-based segmentation method for the generation of higher quality images. GANs are also utilized in [47], in order to generate X-rays data sets from 307 images of four different types. The method employed in [48] utilizes auxiliary classifier generative adversarial network (AC-GAN) to generate COVID-19 CT scans. Then, the authors of [48] compare their approach against competing DL models using transfer learning. The authors of [49] introduce a Mean Teacher plus Transfer GAN (MTT-GAN) model, in order to generate COVID-19 chest X-ray images of high quality. Inception-Augmentation GAN (IAGAN), a semi-supervised GAN-based augmentation method, is introduced in [50], in order to improve the detection capability of pneumonia and COVID-19 in chest X-ray images. The authors of [51] present the QuNet model to classify the COVID-19-infected patients by using X-ray images. In [52] an Enhanced Super Resolution GAN (ESRGAN) is used in order to improve the CT scan quality, before feeding it to a Siamese Capsule network. Additionally, in [53], MESRGAN+ is derived by implementing a connected nonlinear mapping between noise-contaminated low-resolution input images and deblurred and denoised HR images using the building blocks of GAN. A summarizing overview of the main literature on GAN-based models for COVID-19 detection is provided in Table 1.

**Table 1:** Synoptic view of recent work on GAN-based COVID-19 detection

Ref	Approach	Goal	Training class
[45]	CovidGAN	X-ray synthetic augmentation	COVID
[47]	GAN +transfer learning	X-ray synthetic augmentation	Normal+COVID+Pneumonia
[46]	Dense GAN+ U-Net	Enhancing the quality of CT	COVID
[54]	Convolutional GAN	X-ray synthetic augmentation	Normal+COVID+Pneumonia
[48]	AC-GAN	CT synthetic augmentation	COVID
[49]	MTT-GAN	X-ray synthetic augmentation	COVID
[50]	IAGAN	X-ray synthetic augmentation	COVID
[51]	GAN	X-ray synthetic augmentation	Normal+COVID+Pneumonia
[52]	Siamese-CapsNet	Image resolution enhancement	COVID+NON-COVID
[53]	GAN	Image resolution enhancement	COVID+NON-COVID
**This contribution**	BiGAN & CycleGAN	CT Classification	COVID

Overall, differently from the proposed work, all these approaches do not exploit the use of an additional encoder (BiGAN) or a second generator (CycleGAN) that, ideally, learn to invert the mapping performed by the first generator. We argue that a trained BiGAN encoder and a pair of generators/discriminators, respectively, could provide useful feature representation for related tasks of scan classification. Although the increased computational cost with respect to a standard GAN architecture, we can expect that, by considering the performance-vs.-complexity tradeoff, the proposed method can represent a promising approach for the robust classification of the COVID-19 disease from unlabeled CT scans.

### Paper contributions and roadmap

Motivated by the performed review, in this contribution, we aim at exploiting how and at which extend the hidden features learned by weakly supervised BiGAN [55] and CycleGAN [56] models could be effectively exploited for robust classification of COVID-19 diseases from unlabeled CT scans. In fact, both BiGAN and CycleGAN allow to efficiently extract meaningful features of the target class from the encoded vector, which can be successfully used to construct a statistical representation suitable to detect scans of COVID-19 patients from the others. Specifically, the main contributions of this paper are the following ones:

- We exploit the kernel density estimation (KDE) approach for deploying an inference method that utilizes the hidden features generated during the weakly supervised training of BiGANs and CycleGANs for estimating the underlying PDF of CT scans of COVID-19 patients, namely the target COVID-PDF. Afterward, in the test phase, the trained BiGAN/CycleGAN encoder is used for extracting the hidden features from the corresponding COVID/Non-COVID CT test scan, and, then, the distance among the target COVID-PDF and the corresponding PDF of the hidden features extracted from each test image is used for binary classification. For this purpose, a suitably designed binary detector is employed, which is equipped with a tunable decision threshold;
- We numerically evaluate the sensitivity of the achieved accuracies, test times and training times of the implemented BiGANs and CycleGANs on the employed training loss functions and inter-PDF distance metrics. The tested loss training functions are the cross-entropy (CE), least squares (LS) and Wasserstein (W) ones, while the Euclidean, Kullback-Leibler (KL) divergence, Correlation and Jensen-Shannon (JS) divergence are tested as inter-PDF distance metrics;
- The training of the BiGAN and CycleGAN models is, by design, of weakly supervised type. Hence, as a final contribution, we compare the attained BiGAN and CycleGAN performance against the corresponding ones of some recently published methods [38] and [57], which exploit the encoders of unsupervised trained CAEs as feature extractors. In this regard, we anticipate that the implemented CycleGAN model achieves the highest test accuracy, while the tested CAE models attain the lowest test and training times. The corresponding accuracies, test times and training times of the implemented BiGAN models fall somewhat in the middle.

To the best of our knowledge, the exploitation of a KDE estimation of the target COVID-PDF from the feature encoded by the BiGAN and CycleGAN for the classification of COVID/Non-COVID CT scans is novel and not yet investigated in the current literature.

The rest of the paper is organized as follows. In Sect. 2, we describe, at first, the employed training/test data sets, the implemented BiGAN and CycleGAN models and the related training loss functions. Afterward, we present the proposed KDE-based method for test inference. Section 3 is devoted to the presentation of the obtained numerical results and related performance comparisons. Finally, the conclusive Sect. 4 summarizes the main results of the paper and highlights some possible hints for future research.

## Material and solving method

This section describes the used data sets and the implemented BiGAN and CycleGAN-based architectures for feature extraction, together with the companion PDF-based approach pursued for binary classification of the test images.

### Training and testing data sets

**Fig. 1:**
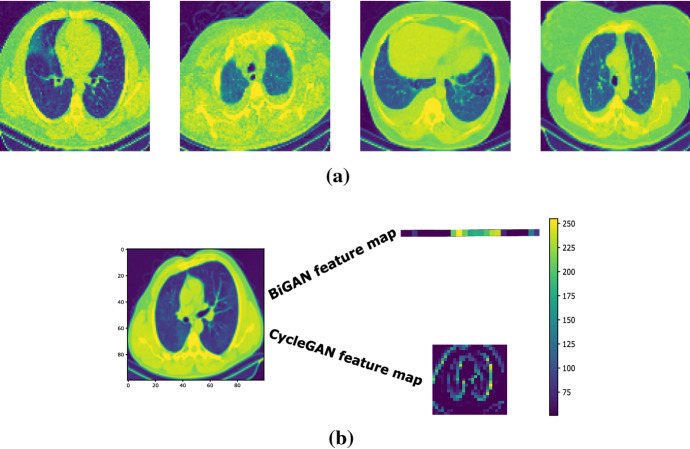
**a** Four representative samples of lung CT scans from the training sets of size ($100\times 100$). **b** Representative samples of lung CT feature maps extracted by the implemented BiGANs and CycleGAN

We selected 1000 COVID-19 CT scans related to 500 (anonymous) patients from several multiple open-access data sets [58], in order to generate the training data set. However, before training, a pre-processing step has been carried out, in which the borders of all CT scans have been cropped and all the gray-scale images have been resized to $100 \times 100$ pixels, in order to achieve a suitable processing complexity-vs.-image resolution trade-off. Finally, the per-pixel mean of each image has been evaluated and subtracted. In the sequel, we will indicate as *y* (resp. *Y*) an input COVID-19 training image (resp., the set of the COVID-19 training images). For illustrative purposes, Fig. 1a reports four examples of COVID-19 training images. Since the considered CAE models require unsupervised learning, only the set *Y* is utilized for their training. However, for both BiGAN and CycleGAN models that rely on weakly supervised learning [55, 56], a second set *X* composed by 1000 input features (also referred to as latent feature maps) has been generated for their training. Specifically, according to [55], each training input feature $x \in X$ has been generated by randomly sampling (in an independent and identically distributed way) from a continuous probability density function, which is evenly distributed over the interval $[-100,100]$. The random procedure adopted for generating the training features assures that the elements of the resulting training sets *X* and *Y* are unpaired, as required by the weakly supervised training of BiGAN and CycleGAN models [55, 56]. In this regard, we anticipate also that, although, in our tests, the feature maps $\left\{ \hat{x}\right\}$ extracted by each model have the same size of the corresponding input feature maps $\left\{ {x}\right\}$, nevertheless, their size varies from model to model (see the 6th column of Table 8). For illustrative purposes, Fig. 1b reports two feature maps extracted from the implemented BiGAN and CycleGAN models.

Finally, we point out that CT scans for testing have been randomly sampled from two data sets [58, 59], which embrace: (i) 500 CT slices of COVID-19 images (different from those used for the training); and, (ii) 500 additional CT scans, which cover normal cases, pneumonia cases and three types of lung cancer (namely, adenocarcinoma, large-cell carcinoma and squamous-cell carcinoma).

### The considered encoder-equipped GAN models

In order to perform classification based on the compressed versions of images (feature representations), BiGANs and CycleGANs are of interest, because they allow to efficiently extract the encoded features of the target class. In the following, we shortly present the implemented models.

#### Cross-entropy BiGANs for feature extraction

**Fig. 2:**
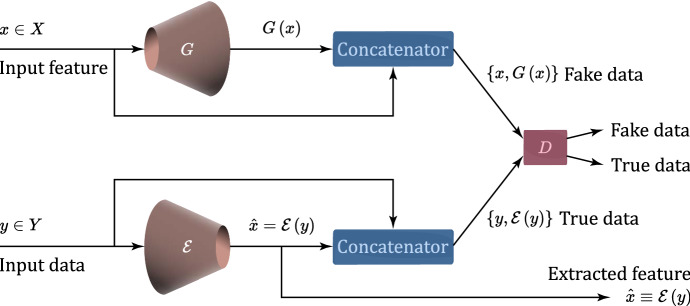
Implemented training scheme of BiGAN [55] for feature extraction. *x*: input feature; *y*: input data; *X*: feature space; *Y*: data space; *G*: Generator; $\mathcal {E}$: Encoder; *D*: Discriminator; $\hat{x}$: predicted and extracted feature

BiGANs offer a framework for weakly supervised feature learning. A BiGAN includes a GAN’s generator *G*, and an encoder $\mathcal {E}$, which maps input data $y \in Y$ (i.e., COVID-19 images, in our framework) to feature representations $\mathcal {E} \equiv \hat{x}$ [43]. The BiGAN discriminator, *D*, discriminates not only in the data space (i.e., *y*-vs.-$G\left( x\right)$), but jointly in the latent and data spaces (i.e., $\left\{ y, \mathcal {E}\left( y\right) \right\} \text {-vs.-}\left\{ x, G\left( x\right) \right\}$) versus (*G*(*z*); *z*), where the latent component is either an encoder output: $\mathcal {E}(y)$ or a generator input: *x* (see Fig. 2).

The BiGAN encoder, $\mathcal {E}$, aims to learn to invert the mapping performed by the generator *G* [55]. Neither module can directly communicate with the other; the encoder cannot see the generator outputs and the generator cannot see the encoder outputs.

The final goal of both encoder and generator is to fool the BiGAN discriminator, *D* [55]. For this purpose, the BiGAN encoder learns to predict features $\hat{x}$ from input data *y*. Since previous work on BiGAN proved that the extracted features capture semantic attributes of the input data, we argue that a trained BiGAN encoder could provide useful feature representation for related semantic tasks. Toward this end, the BiGAN negative-log-likelihood training objective is defined as follows (see [55] for major details):

1 $$\begin{aligned} \begin{aligned} \underset{G,\mathcal {E}}{\min } \, \underset{D}{\max }\ V\!\left( D,\mathcal {E},G\right) =&\, E_{y \sim p_{y}} \underset{\log D\left( y,\mathcal {E} \left( y \right) \right) }{\underbrace{{E_{x\sim p_{\mathcal {E}}\left( .\mid y \right) }\left[ \log D\!\left( x,y \right) \right] }}} \\&+E_{x\sim p_{x} }\underset{\log \left( 1-D\left( G \left( x \right) , x \right) \right) }{\underbrace{E_{y\sim p_{G}\left( .\mid x \right) } \left[ 1 - \log D\!\left( x,y \right) \right] }}. \end{aligned} \end{aligned}$$

While BiGANs retain many properties of GANs, they also guarantee that *G* and $\mathcal {E}$ are each other’s inverse at the global optimum. BiGAN training is carried out by using an optimizer for training the parameters $\ {\theta }_{D}$, $\ {\theta }_{G}$, and $\ {\theta }_{\mathcal {E}}$ of modules *D*, *G* and $\mathcal {E}$, respectively. Training consists of performing one or more steps in the positive gradient direction to update the discriminator parameters $\ {\theta }_{D}$. A step in the negative gradient direction is, then, performed, in order to update the encoder and generator parameters $\ {\theta }_{\mathcal {E}}$ and $\ {\theta }_{G}$. In the following sections, we refer to the BiGAN trained according to Eq. (1) as Cross-Entropy BiGAN (CE-BiGAN). The architecture of the actually implemented BiGAN is detailed in Table 2. In our tests, the size of the extracted latent vector $\mathcal {E}\left( y\right)$ in Fig.  2 is set to 1024. All the activation functions are leaky ReLUs with slope of 0.1, barring the last layer of the generator, in which the hyperbolic tangent activation function is used (see Table 2).

**Table 2:** The implemented BiGAN architecture. Conv: Convolution; ConvTr: Transposed Convolution; BN: Batch Normalization; LR: Leaky ReLU; DR: Dropout

(a) Generator		
Noise	$1024\times 1$	
Dense+BN+LR	$80000\times 1$	
Reshape	$25\times 25\times 128$	
ConvTr+BN+LR	$50\times 50\times 128$	
ConvTr+BN+LR	$50\times 50\times 128$	
ConvTr+BN+LR	$100\times 100\times 256$	
ConvTr+BN+Tanh	$100\times 100\times 1$	

(b) Encoder		
Conv+BN+LR	$100\times 100\times 1$	
Conv+BN+LR	$100\times 100\times 256$	
Conv+BN+LR	$50\times 50\times 128$	
Conv+BN+LR	$50\times 50\times 128$	
Conv+BN+LR	$25\times 25\times 128$	
Flatten	$80000\times 1$	
Dense+BN+LR	$1024\times 1$	

(c) Discriminator		
	Input1	$100\times 100\times 1$
	Conv+LR+DR	$100\times 100\times 256$
	Conv+LR+DR	$50\times 50\times 128$
	Conv+LR+DR	$25\times 25\times 128$
	Conv+LR+DR	$25\times 25\times 128$
	Flatten	$80000\times 1$
	Input2	$1024\times 1$
	Dense+DR	$512\times 1$
	Dense	$512\times 1$
	Output	$512\times 1$
	Concatenate	Flatten + Output
	Dense+DR	$1024\times 1$
CE-BiGAN	Dense+Sigmoid	1
W-BiGAN	Dense+Linear	1
LS-BiGAN	Dense+Linear	1

#### Least-squares BiGANs for feature extraction

Least-squares generative adversarial networks (LSGANs) adopt the least squares loss function for training [60]. The authors of [60] point out two advantages of LSGANs over standard CE-GANs. First, LSGANs are capable of generating images of higher-quality than CE-GANs. Second, LSGANs also exhibit more stable performance during the learning process. In fact, since a CE-GAN discriminator typically adopts the sigmoid cross-entropy loss function, when the generator is updated, vanishing gradient may happen for samples on the correct side of the decision boundary, which are still far from the real data [60]. LSGANs attempt to bypass this problem by using the following least squares-based training loss function:

2 $$\begin{aligned} \begin{aligned} \underset{G}{\text {min}}\ V_{LSGAN} \left( G\right) \triangleq&\; \frac{1}{2}E_{x\sim p_{x}}\left[ \left( D \left( G \left( x \right) , x \right) - c \right) ^2 \right] , \\[2ex] \underset{D,\mathcal {E}}{\min }\ V_{LSGAN} \left( D,\mathcal {E}\right) \triangleq&\; \frac{1}{2} \left\{ E_{y\sim p_{y}} \left[ \left( D \left( y ,\mathcal {E} \left( y\right) \right) - a \right) ^2 \right] \right. \\&\left. + E_{x\sim p_{x} } \left[ \left( D \left( G \left( x \right) , x \right) - b \right) ^2 \right] \right\} , \end{aligned} \end{aligned}$$

where *a* and *b* are the labels for true data and fakes, while *c* indicates the value that *G* wants *D* to believe for fake data [60]. As suggested in [60], in our test, we set $a=c=1$, and $b=0$ where 0-1 is binary labeling scheme used for fake-true data. We apply the loss function of Eq. (2) together with the linear activation function in the last layer of the discriminator of Fig. 2. The architecture of the implemented BiGAN is still the one of Table 2.

#### Wasserstein BiGANs for feature extraction

Wasserstein GANs (WGANs) [61] generate loss functions with better characteristics than the cross-entropy original GANs by using the Wasserstein distance. For this purpose, the authors of [61] impose weight clipping by requiring that the discriminator (called *critic* in their paper) falls in the 1-Lipschitz space. Accordingly, the loss function of a Wasserstein BiGAN (W-BiGAN) is defined as in [61]:

3 $$\begin{aligned} \mathcal {L}_{\text {W-BiGAN}} = \frac{1}{M} \sum _{y \sim p_y} D \left( y, \mathcal {E} \left( y \right) \right) - \frac{1}{M} \sum _{x\sim p_x} D \left( G \left( x \right) , x \right) , \end{aligned}$$

where $M \ge 1$ is the number of terms in each summation. Ad pointed out in [62], the r.h.s. of (3) provides, indeed, a reasonable good computable approximation of the actual Wasserstein distance. Unlike the original BiGAN where *D* is a 0/1 classifier estimating the *a posteriori* probability that its input is a true data, in the Wasserstein BiGAN (W-BiGAN), *D* is a regressor, which estimates the *trueness* score of its input. In terms of implementation, the scalar output of *D* in the original BiGAN uses the sigmoid nonlinearity, while that of the W-BiGAN is linear. The Wasserstein loss in Eq. (3) is the difference of the trueness scores of true and fake samples. *D* is trained to maximize this difference, while *G* is trained to minimize it. *D* wants that its output: $D(y,\mathcal {E}(y)$ is higher for true samples *y* than for the generated fake samples: *D*(*G*(*x*), *x*), while *G* aims at the opposite. Due to the interactions between weight constraints and cost function, WGAN optimization process may result in either vanishing or exploding gradients if the clipping threshold calibration is not suitably tuned [62]. After several validation trials, we set the weight clipping value to 0.01 and normalize the norm of error gradient vector to 10. The same architecture of BiGAN of Table 2 is utilized under the training loss function in Eq. (3), with the linear activation function in the last layer of the W-BiGAN discriminator.

#### CycleGANs for feature extraction

**Fig. 3:**
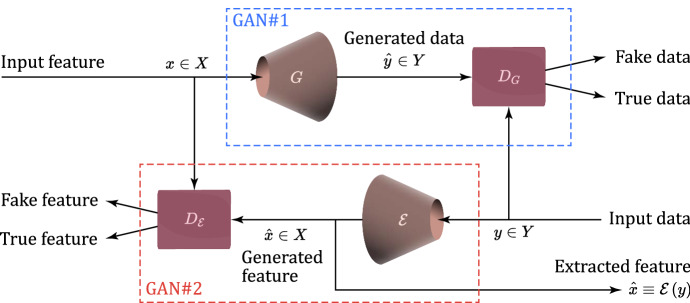
Implemented scheme of CycleGAN for feature extraction. *x*: Input feature; *y*: Input data; $\hat{x}$: Generated and extracted feature; $\hat{y}$: Generated data; *X*: Feature space; *Y*: Data space; *G*: Generator; $\mathcal {E}$: Encoder; $D_G$: Generator’s discriminator; $D_{\mathcal {E}}$: Encoder’s discriminator

An input image that is transformed by a CycleGAN [56] can retain fine details, so to closely reproduce the structure of the input image. CycleGAN explores the unpaired style transfer paradigm, in which the model attempts to learn stylistic differences between sources and targets without explicitly pairing input to output [63]. As sketched in Fig. 3, a CycleGAN has two generators, *G* and $\mathcal {E}$, such that $G:X\longrightarrow Y$ and $\mathcal {E}:Y\longrightarrow X$. Ideally, *G* and $\mathcal {E}$ should be the inverse of each other, so to implement one-to-one bijection. The authors of [56] train simultaneously both the generators *G* and $\mathcal {E}$ under both adversarial and cycle consistency losses, so to encourage $\mathcal {E} \left( G \left( x\right) \right) \cong x$ and $G \left( \mathcal {E} \left( y\right) \right) \cong y$. A CycleGAN is typically equipped with two discriminators $D_G$ and $D_\mathcal {E}$ which are paired to the corresponding generators *G* and $\mathcal {E}$, respectively. In [56], it is argued that a pair of generators/discriminators could learn the best possible translation from the source domain *Y* (or *X*) to the target domain *X* (or *Y*). The overall cycle consistency loss $\mathcal {L}_{\text {Cyc}}$ ensures that the reconstruction of the original input from the generated output is as close as possible, and it is defined as in [56]:

4 $$\begin{aligned} \mathcal {L}_{Cyc} \left( G,\mathcal {E}\right) = E_{x\sim p_x}\left\| \mathcal {E} \left( G \left( x \right) \right) - x \right\| _{1}+E_{y\sim p_y}\left\| G \left( \mathcal {E} \left( y \right) \right) -y \right\| _{1}. \end{aligned}$$

Afterward, the overall objective of a CycleGAN is a weighted sum of the adversarial losses: $\mathcal {L}_{\text {GAN1}}$ and $\mathcal {L}_{\text {GAN2}}$ and the cycle consistency loss $\mathcal {L}_{\text {Cyc}}$, and, then, it reads as in:

5 $$\begin{aligned} \mathcal {L}_{\text {CycleGAN}} \left( G,\mathcal {E},D_{G} ,D_{\mathcal {E}}\right) \triangleq \mathcal {L}_{\text {GAN1}} \left( G,D_{G}\right) + \mathcal {L}_{\text {GAN2}} \left( \mathcal {E},D_{\mathcal {E}}\right) + \lambda \, \mathcal {L}_{\text {Cyc}} \left( G,\mathcal {E}\right) . \end{aligned}$$

In our tests, $\lambda = 0.1$ and the Wasserstein loss function is employed to implement both the adversarial losses $\mathcal {L}_{\text {GAN1}}$ and $\mathcal {L}_{\text {GAN2}}$ in Eq. (5). The implemented CycleGAN is sketched in Fig. 3. In this regard, we stress that we use it for feature extraction (see Fig. 3). The size of the extracted features is reported in the 6th column of Table 8.

### The pursued KDE-based inference approach

In order to estimate the probability density function (PDF) of the extracted hidden features, generated by (previously described) GAN-based models, the first step is to choose among parametric-vs.-non-parametric methods. Due to the fact that we have no *a priori* information about the actual shape of the PDF and we want to avoid bias effects, we choose a non-parametric estimate. For this purpose, we select the kernel density estimation (KDE) method due to its efficiency and expected performance [64]. To describe the KDE, we first illustrate it for the simple case of a univariate PDF. Hence, let us consider a set of *n* real numbers: $x_i$ for $i = 1, ..., n$, drawn from a (hidden) Random Variable (RV) ${\varvec{X}}$, which possess an unknown PDF, $f_X\left( x\right)$, to be estimated. Hence, the KDE estimate $\bar{f}_x \left( x\right)$ of $f_x \left( x\right)$ is defined as:

6 $$\begin{aligned} \bar{f}_X(x) \triangleq \frac{1}{\alpha } \sum _{i=1}^{n} K \left( \frac{x-x_i}{h} \right) . \end{aligned}$$

The constant $\alpha$ is a normalization factor, which guarantees that the area under the curve $\bar{f}_X\left( x\right) , x \in \mathbb {R}$, is unit valued. The kernel function, *K*(.), is used as an interpolating function to build the PDF estimate. Although different kernels can be used, according to [64], we consider the Gaussian one, i.e., $K(x) = e^{-x^2}$. The parameter *h* in Eq. (6) is the kernel bandwidth, which is used to set the width of the kernel. It controls the size of the receptive field of the kernel. Since our inference method is based on the evaluation of the distances between actual and target PDFs, we have numerically ascertained that the impact of *h* is minor. Hence, we set the bandwidth to the unit.

The target COVID-PDF is evaluated by applying Eq. (6) to the average of all the extracted feature vectors obtained by the encoders of the considered architectures for all the training images.

### Exploiting hidden features for test classification

**Fig. 4:**
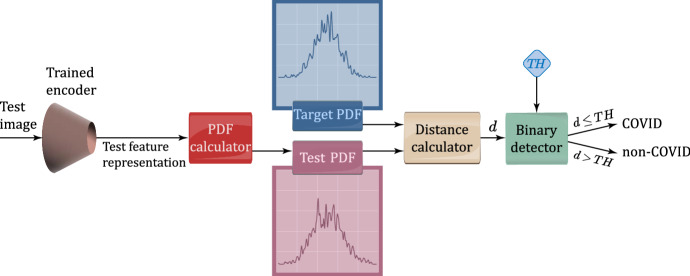
Proposed inference mechanism for binary classification of test images

After training on COVID-19 through the BiGANs and CycleGAN, we evaluate the proposed classification method. Using the procedure shown in Fig. 4, we classify each test image. To this end, we only deal with the encoders of BiGANs and the first generator’s encoder of CycleGAN. In order to accomplish this, each COVID-19 test image is fed to the trained encoder and its corresponding hidden feature vector is extracted. After computing the PDF of the test feature vector through KDE, the distance *d* between the target and test PDFs is evaluated and given as input to a binary threshold detector (see the lost block of Fig. 4). This last generates the final COVID/non-COVID decision on the corresponding input image.

**Fig. 5:**
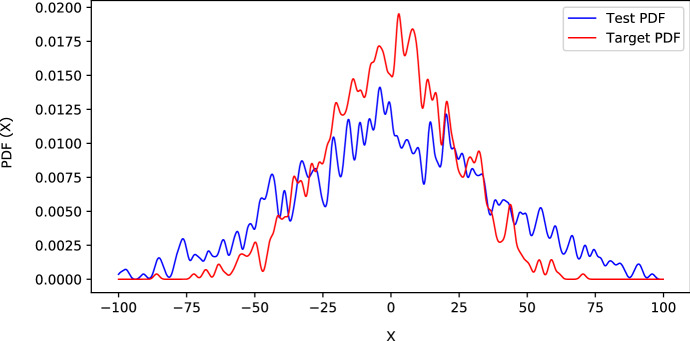
Instances of target and test PDFs

Figure 5 shows two examples of attained target and test PDFs.

*Used distance metrics:* The target COVID-PDF and the test PDF are compared by using a suitable distance in the latent space. In order to formally introduce the considered inter-PDF distances, let $\mathbf {P}$ and $\mathbf {Q}$ be two equal-size probability column vectors and let: $\mathbf {M} \triangleq \left( \mathbf {P} + \mathbf {Q}\right) /2$ be the corresponding mean distribution vector. Hence, the considered Euclidean, KL, Correlation and Jensen-Shannon distances are formally defined in Table 3, where $p_i$ (resp. $q_i$) indicates the *i*-th entry of $\mathbf {P}$ (resp. $\mathbf {Q}$) and the *T* superscript means vector transposition.

**Table 3:** Considered inter-PDF distances

Considered metric distance	Formula
Euclidean	$\left\| \mathbf {P}-\mathbf {Q} \right\| _{2}$
Kullback-Leibler divergence	$\sum \limits_{i=1}^{n}p_{i}\text {log}\left( \frac{p_{i}}{q_{i}} \right)$
Correlation	$1-\frac{\left( \mathbf {P}-\mathbf {\bar{P}} \right) ^T\left( \mathbf {Q}-\mathbf {\bar{Q}} \right) }{\left\| \left( \mathbf {P}-\mathbf {\bar{P}} \right) \right\| _{2} \, \left\| \left( \mathbf {Q}-\mathbf {\bar{Q}} \right) \right\| _{2}}$
Jensen-Shannon	$\sqrt{\frac{KL\left( \mathbf {P}\parallel \mathbf {M} \right) +KL\left( \mathbf { Q}\parallel \mathbf {M} \right) }{2}}$

*Setting of the decision threshold:* The decision threshold for each considered distance is set by evaluating the PDFs of all training images. Then, we numerically calculate the distance between the target COVID-PDF and each training image PDF and set the threshold $T H$ to the obtained maximum distance value. So doing, the attained value of the threshold is automatically tuned to the statistical properties of both the underlying target PDF and used distance metric. In this regard, we anticipate that, in our tests of Sect. 3, the (numerically evaluated) values of the tuned decision thresholds typically range from 0.06 to 0.6.

## Comparative numerical results and discussion

The main goal of this section is twofold. First, after describing the experimental setup and the adopted performance indexes, we discuss the sensitivity of the training and test performance of the implemented BiGAN and CycleGAN models on the considered training loss functions and inter-PDF distance metrics. Second, we present the accuracy-vs.-test time-vs.-training time performance of the implemented BiGAN and CycleGAN models and, then, compare them against the corresponding ones of the CAE-based models recently presented in [38] and [57].

### Considered performance metrics

The considered performance metrics for the carried out binary classification tasks are based on the True Positive (TP), True Negative (TN), False Positive (FP), and False Negative (FN) assignments. The meaning of these outcomes is detailed in Table 4. They can be represented in a compact form as the four elements of the resulting confusion matrix [65].

**Table 4:** Main outcomes in binary classification

Taxonomy	Description
True Positive (TP)	COVID-19 image classified as COVID-19
True Negative (TN)	Non-COVID-19 image classified as non-COVID-19
False Positive (FP)	Non-COVID-19 image classified as COVID-19
False Negative (FN)	COVID-19 image classified as non-COVID-19

The main performance metrics can be derived by a combination of these items [65]. In the following sections, we consider accuracy, recall, precision, F1-score, area under the receiver operating characteristic (ROC) curve (AUC) as affiliated performance indexes. Formal definitions of these indexes are given in Table 5.

**Table 5:** Performance metrics for binary classification [65]

Performance index	Formula
Recall	$TP/(TP+FN)$
Precision	$TP/(TP+FP)$
F-score	$2\,TP/(2\,TP+FP+FN)$
Accuracy	$(TP+TN)/(TP+FN+FP+TN)$

### Experimental setup

All the numerical tests have been carried out on a PC equipped with: (i) an AMD Ryzen 9 5900X 12-Core 3.7 GHz processor; (ii) two GeForce RTX 3080 graphics cards; and (iii) 128 GB RAM.

The iterative solving algorithm used for the training of the implemented CE-BiGAN, LS-BiGAN and LS-CycleGAN models is Adam [66], while the W-BiGAN is trained by using the RMSprop solver with clipping threshold set to 0.01 [61]. The hyper-parameters of all implemented solvers have been optimized through validation trials, and their main optimized values are reported in Table 6. Mini-batches of size of 16 have been utilized for model training under all implemented solvers.

**Table 6:** Implemented solvers for the optimization and related hyper-parameter tuning. LR: Learning Rate; WclipV: Weight clip Value; GclipN: Gradient clip Norm

Model	Solver	LR	$\varvec{\beta _1}$	WclipV	GclipN
CE-BiGAN	Adam	$10^{-4}$	0.1	–	–
W-BiGAN	RMSprop	$10^{-5}$	–	0.01	10
LS-BiGAN	Adam	$10^{-4}$	0.1	–	10
LS-CycleGAN	Adam	$10^{-4}$	0.35	–	–

### Comparison of the simulated training loss curves

According to [55, 60, 61], a training iteration of each implemented BiGAN and CycleGAN model embraces $m \ge 1$ gradient-based steps for the optimization of the underlying discriminators, which are followed by a single step for the optimization of the corresponding generators. We have numerically ascertained that, in our framework, $m=1$ (resp., $m=5$) is suitable for the training of the CE-BiGAN, LS-BiGAN and LS-CycleGAN models (resp., for the training of the W-BiGAN model).

**Fig. 6:**
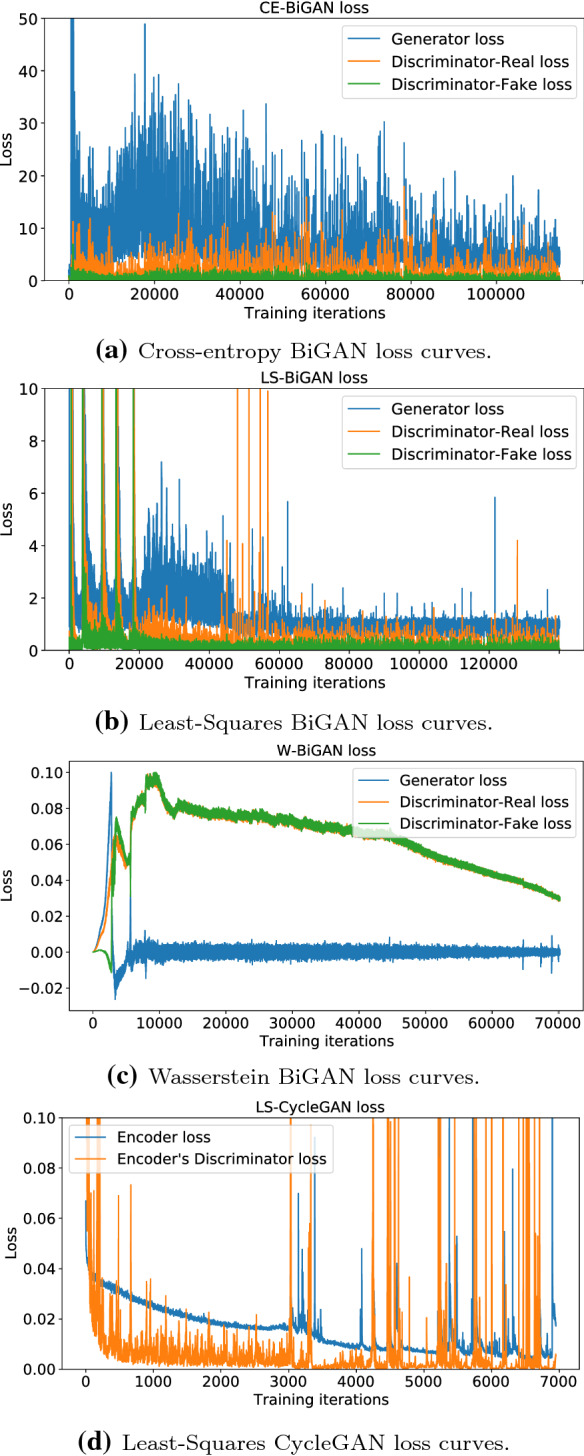
Training loss curves of the considered approaches

The attained loss curves are reported in Fig.  6. Regarding the BiGAN model, a comparative examination of the training curves of Figs. 6a–c points out that the Wasserstein (resp., Cross-Entropy) loss function gives rise to the most (resp., less) stable behavior during the overall training phase, with the behavior of the least-squares loss function falling somewhat in the middle. This conclusion is also supported by the following additional two remarks. First, a comparative view of the entries in the second column of Table 11 unveils that the number of training iterations needed for achieving the convergence is the highest (resp., the lowest) one for the CE-BiGAN (resp., the W-BiGAN), with the ranking of the LS-BiGAN still falling in the middle. In detail, as it could be expected, the two discriminator losses of Fig. 6c nearly overlap, so that the resulting generator loss fluctuates around zero and asymptotically vanishes. Second, the results reported in Table 7 show that, under each checked distance metric, the corresponding test accuracy of the W-BiGAN is the highest one, although the relative gaps with respect to the competing CE-BiGAN and LS-BiGAN models are not so impressive. However, we have numerically ascertained that, at least under the considered training dataset, the least-squares loss function gives rise to the most stable behavior in the training phase of the implemented CycleGAN (see the plots of Fig. 6d). So, in the following sections, we directly focus on the LS-CycleGAN model.

### Performance robustness with respect to the distance metrics

The impact of the considered distance metrics on the performance indexes of Table 5 in the test phase may be evaluated through a comparative view of the entries of Table 7. In this regard, three main conclusions may be drawn. First, the test performance of all models is quite robust with respect to the choice of the distance metric used for implementing the classifier of Fig. 4. Specifically, the resulting accuracy gaps over the full spectrum of checked model-vs.-distance settings are, indeed, limited up to 5.7%. Second, the accuracies of the CE-BiGAN and LS-BiGAN (resp., W-BiGAN and LS-CycleGAN) models attain their corresponding maxima under the correlation (resp., Jensen-Shannon) distance metric. Third, the highest test accuracy is obtained by the LS-CycleGAN model combined with the Jensen-Shannon distance.

**Fig. 7:**
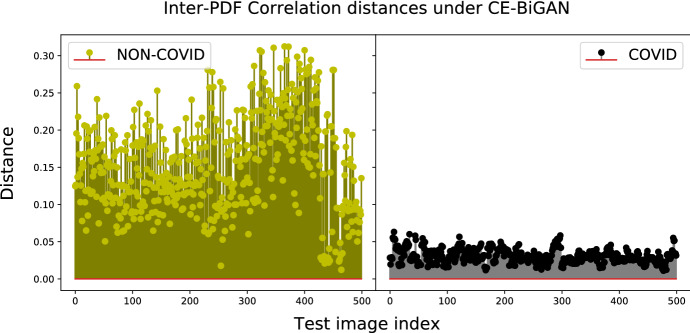
Correlation distances between test-PDFs and COVID-PDF of CE-BiGAN; Threshold: $T H = 0.06$

**Fig. 8:**
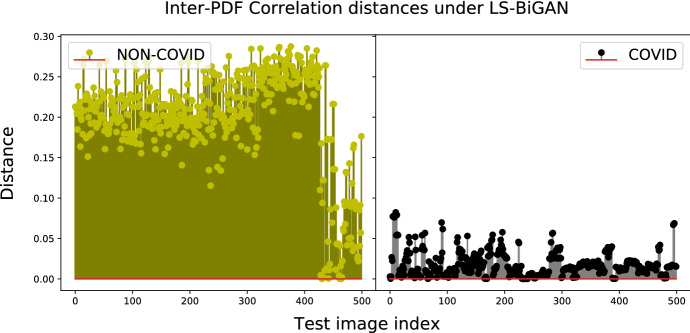
Correlation distances between test-PDFs and COVID-PDF under the LS-BiGAN model; Threshold: $T H = 0.08$

**Fig. 9:**
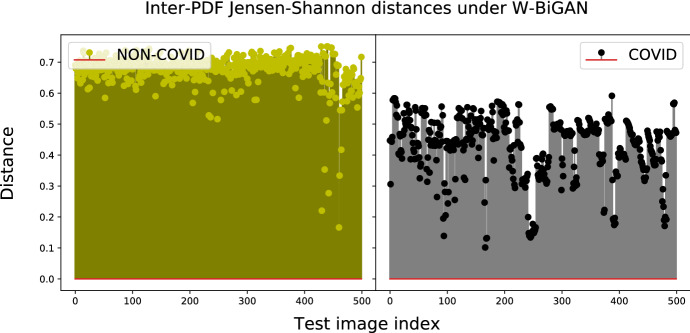
Jensen-Shannon distances between test-PDFs and COVID-PDF under the W-BiGAN model; Threshold: $T H = 0.585$

**Fig. 10:**
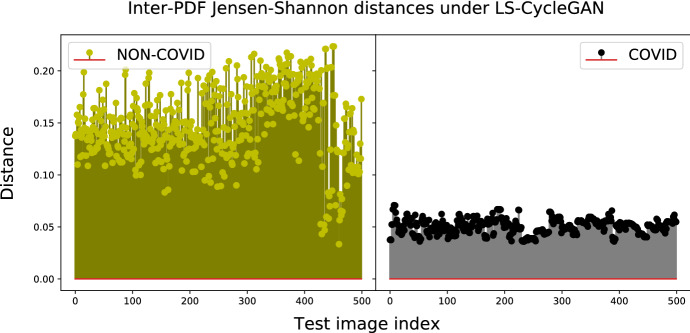
Jensen-Shannon distances between test-PDFs and COVID-PDF under the LS-CycleGAN model; Threshold: $T H = 0.07$

**Fig. 11:**
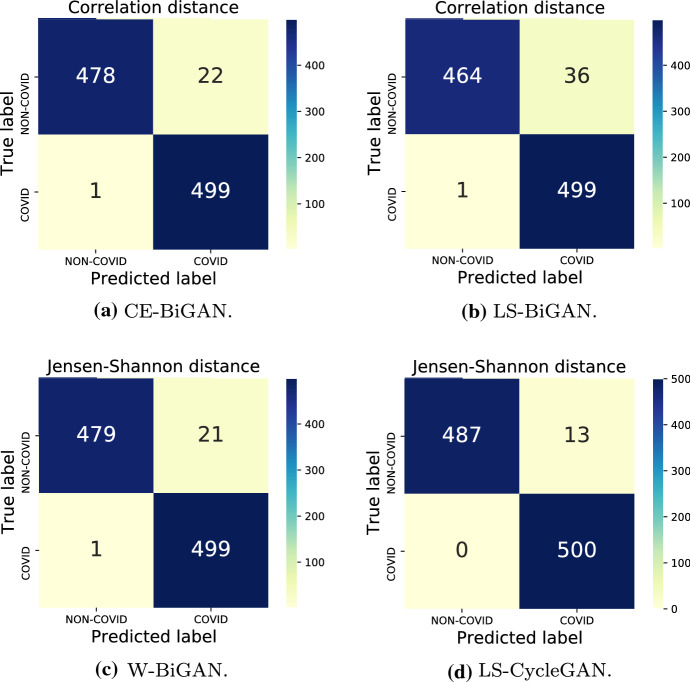
Confusion matrices for the best setting of model/distance metric

The numerically evaluated distance spectra between the test and target COVID PDFs of the checked models under their corresponding best distance metrics are drawn in Figs. 7, 8, 9 and 10, while the associated confusion matrices are reported in Fig. 11.

The reported distance spectra corroborate the conclusion that the gaps between the accuracy performance of the best-checked models are, indeed, limited, with a slight superiority of the LS-CycleGAN model combined with the Jensen-Shannon distance (see Fig. 11).

**Fig. 12:**
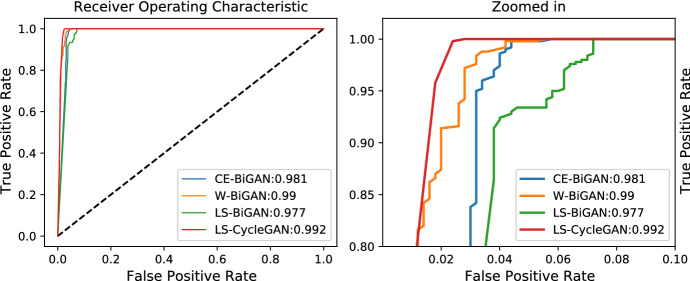
ROCs of the tested models under the corresponding best distance metrics

This conclusion is further supported by the ROC curves of Fig. 12 and the associated AUC values (see the legend of Fig. 12). These curves confirm, indeed, that the LS-CycleGAN model combined with the Jensen-Shannon distance metric (resp., the LS-BiGAN model combined with the Correlation distance metric) attains the highest (resp., lowest) AUC value of 0.992 (resp., 0.977).

**Table 7:** Model performance under different distance metrics

Model	Distance	Accuracy	Precision	Recall	F1-score	Test CTs
CE-BiGAN						
	Euclidean	0.9760	0.9760	0.9765	0.9760	1000
	Correlation	**0.9770**	0.9770	0.9778	0.9770	1000
	KL divergence	0.9740	0.9740	0.9746	0.9740	1000
	Jensen-Shannon	0.9720	0.9720	0.9728	0.9720	1000
LS-BiGAN						
	Euclidean	0.9490	0.9490	0.9492	0.9490	1000
	Correlation	**0.9640**	0.9630	0.9653	0.9630	1000
	KL divergence	0.9560	0.9560	0.9567	0.9560	1000
	Jensen-Shannon	0.9300	0.9300	0.9314	0.9299	1000
W-BiGAN						
	Euclidean	0.9770	0.9780	0.9781	0.9780	1000
	Correlation	0.9770	0.9770	0.0772	0.9770	1000
	KL divergence	0.9770	0.9770	0.9772	0.9770	1000
	Jensen-Shannon	**0.9780**	0.9780	0.9788	0.9780	1000
LS-CycleGAN						
	Euclidean	0.9760	0.9760	0.9766	0.9760	1000
	Correlation	0.9700	0.9700	0.9711	0.9700	1000
	KL divergence	0.9860	0.9860	0.9863	0.9870	1000
	Jensen-Shannon	**0.9870**	0.9870	0.9873	0.9870	1000

Bold indicates the best result

### Unsupervised-vs.-weakly supervised models: comparative performance

By design, all the considered BiGAN and CycleGAN models require weakly supervised (WS) training (see Sect. 2.1). Hence, it could be of interest to compare their implementation complexity-vs.-training time-vs.- test time-vs.-test accuracy trade-offs against the corresponding ones of the companion models in [38, 57], which have been recently developed in the literature for COVID-19 detection/classification. Like the considered BiGANs and CycleGANs, even the models developed in [38, 57] rely on suitably extracted hidden features for performing distance-based classification. However, unlike the here considered GAN-based models, both the models developed in [38, 57] exploit the encoders of UnSupervised (US)-trained Denoising CAEs (DCAEs) to extract suitable hidden features from COVID-19 input images. Shortly, the extracted hidden features are utilized in [38] for building up suitable target and test histograms, while they are used in [57] for estimating the underlying test and target PDFs. Hereinafter, we refer to the model in [38] (resp., [57]) as the Histogram-Based DCAE (HB-DCAE) (resp., Probability density-Based CAE (PB-CAE)).

The middle columns of Table 8 allow us to compare the main operating settings of the considered WS/US models in terms of sizes of the used input images, numbers of utilized training and test images and sizes of the extracted feature maps. A comparative description of their interior architectures and numbers of trainable parameters (i.e., model sizes) is presented in Table 10, where the $\times 2$ factors account for the fact that a CycleGAN is composed, by design, of two generators and two discriminator nets (see Fig. 2).

**Table 8:** Implemented unsupervised and weakly supervised models for COVID-19 detection. HB-DCAE: Histogram-Based DCAE [38]; PB-CAE: PDF-Based CAE [57]; US: Un-Supervised; WS: Weakly Supervised; TRIM: Number of TRaining IMages; TSIM: Number of TeSt IMages

Model	Model training	Size of the input images	#TRIM	#TSIM	Size of the extract features	Resulting best test accuracy
HB-DCAE	US	$200 \times 300$	1000	1000	$50 \times 75 \times 64$	0.8270
PB-CAE	US	$200 \times 300$	1000	1000	$128 \times 1$	0.7610
CE-BiGAN	WS	$100 \times 100$	1000	1000	$1024 \times 1$	0.9770
W-BiGAN	WS	$100 \times 100$	1000	1000	$1024 \times 1$	0.9780
LS-BiGAN	WS	$100 \times 100$	1000	1000	$1024 \times 1$	0.9640
LS-CycleGAN	WS	$100 \times 100$	1000	1000	$25 \times 25 \times 256$	**0.9870**

Bold indicates the best result

The corresponding performance of the tested models is measured through numerical evaluation of the resulting test accuracies (see the last column of Table 8), together with the number of required training iterations and associated training and test times (see Table 11). In order to guarantee fair accuracy comparisons, the same number (i.e., 1000) of training and test images is utilized in all tests (see the 4th and 5th columns of Table 8). Furthermore, in order to carry out fair comparisons among the evaluated training times, the following exit condition has been applied in all performed training simulations: The training phase of a model is stopped when the best training accuracy over a window of 30 consecutive iterations improves less than $0.1\%$ compared to the corresponding best training accuracy attained over the previous iteration window.

Finally, Table 9 shows some comparisons with other state-of-the-art approaches in the case of supervised COVID/Non-COVID classification, using the same dataset. Specifically, we provide comparisons with famous CNN models such as AlexNet [34], VGG16 [34], ResNet50 [17], and CovidNet-CT [58]. In addition, we also consider the MERSGAN+ proposed in [52, 53], which combines a modified enhanced super-resolution GAN with a Siamese capsule network, the random forest approach proposed in [33] for large-scale screening, and the AI-based system exploiting U-Net architectures introduced in [18].

**Table 9:** Performance evaluation metrics related to some comparisons with other state-of-the-art approaches using the same dataset

Reference	Model	Accuracy	Precision	Recall	F1-score
[34]	AlexNet	0.9060	0.9060	0.9209	0.9052
[34]	VGG16	0.9140	0.9140	0.9266	0.9134
[17]	ResNet50	0.9245	0.9245	0.9359	0.9302
[58]	CovidNet-ct	0.9790	0.9790	0.9714	0.9752
[52, 53]	MERSGAN+	0.9765	0.9820	0.9840	0.9830
[33]	Random forest	0.9004	0.8917	0.9025	0.8971
[18]	AI-system	0.9656	0.9020	0.9710	0.9352

An examination of Table 9 shows that the proposed BiGAN approaches generally outperform the most common supervised classification methods, although the CovidNet-CT [58] and MERSGAN+ [52] ones obtain similar results. On the other hand, the proposed CycleGAN always outperforms all the state-of-the-art approaches.

Overall, the results shown in Table 9 compared to those of Table 8 demonstrate the effectiveness of the proposed methods, since, although these are weakly supervised approaches, they are able to perform the same or better than the supervised ones.

### Performance-vs.-computational complexity tradeoff

**Fig. 13:**
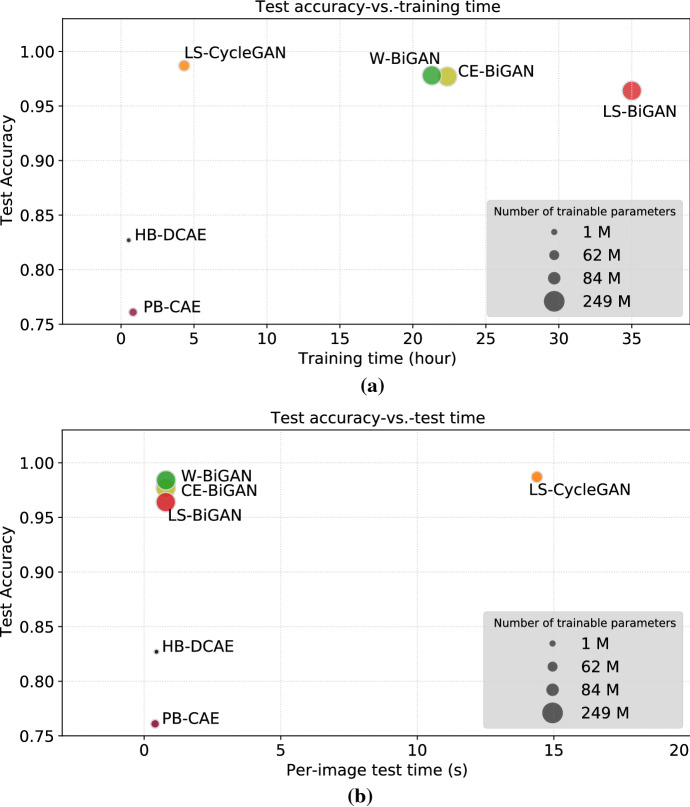
**a** Per-model training time versus test accuracy. **b** Per-model test time versus test accuracy

Figure 13 provides a compact synoptic view of the implementation complexity-vs.-training time-vs.-test time-vs.-accuracy tradeoffs attained by the tested US/WS models. Specifically, in Fig. 13, the diameters of the disk-shaped markers are proportional to the corresponding model sizes (i.e., the number of trainable parameters reported in Table 10).

An examination of Fig. 13 leads to the following insights about the relative merits of the compared models. In terms of test accuracy, the GAN-based models, although present a not negligible training time, outperform the CAE-based ones, with the accuracy of the most performing GAN model (e.g., the LS-CycleGAN one) that is larger than the accuracy of the most performing CAE model (e.g., the HB-DCAE one) of about $16.1\%$ (see also the last column of Table 8). Furthermore, due to their larger learning capability, the GAN-based models are capable to operate on input images whose sizes are smaller than the ones required by the CAE-based models (see the 3rd column of Table 8). We have numerically ascertained that these results are mainly dictated by the US-vs.-WS nature of the tested DL models.

**Table 10:** Details and number of trainable parameters of the considered model architectures. BN: Batch-Normalization; Conv: Convolution; ConvTr: Transposed Convolution

		BiGANs	CycleGAN	HB-DCAE[38]	PB-CAE[57]
*Generator*					
	Conv+BN	–	$21+24 \quad (\times 2)$	–	–
	ConvTr+BN	4+4	$3+3 \quad (\times 2)$	–	–
	Dense	1	$3 \quad (\times 2)$	–	–
	Trainable parameters	82,753,665	$35,264,003 \quad (\times 2)$	–	–
*Discriminator*					
	Conv + BN	4+0	$6+6 \quad (\times 2)$	–	–
	Dense	4	–	–	–
	Trainable parameters	83,826,561	$6,960,321 \quad (\times 2)$	–	–
*Encoder*					
	Conv+BN	4+4	3+3	3+2	3+2
	Dense	1	–	–	1
	Trainable parameters	82,515,072	374,016	376,640	31,096,768

However, in terms of training times, opposite conclusions take place. In fact, as a direct consequence of the major sizes of the GAN-based models compared to the corresponding ones of the CAE-based models, both the number of training iterations and the resulting training times of the implemented BiGAN and CycleGAN models are larger than the corresponding ones of the HB-DCAE and PB-CAE ones. Specifically, the training time of the ‘fastest-to-train’ GAN-based model (e.g., the LS-CycleGAN) is larger than the one of the ‘fastest-to-train’ CAE model (e.g., the HB-DCAE) of about 18.5 times.

**Table 11:** Training/test times of the considered models

Model	Number oftraining iterations	Per-iteration time (s)	Per-image test time (s)
CE-BiGAN	115,000	0.7	0.79
W-BiGAN	70,000	1.08	0.80
LS-BiGAN	140,000	0.9	0.79
LS-CycleGAN	7000	2.23	14.38
HB-DCAE	3125	0.27	0.05
PB-CAE	6250	0.306	0.01

Finally, a similar conclusion holds for the corresponding test times. Specifically, the per-image test times of the ‘fastest-to-test’ GAN-based models (e.g., the Bi-GAN models) are larger than the one of the ‘fastest-to-test’ CAE model (e.g., the PB-CAE) of about 80 times (see the last column of Table 11). In this regard, we have numerically ascertained that the achieved test times are mainly dictated by the sizes of the extracted features. This is also the reason why the test time of the LS-CycleGAN is larger than the ones of the BiGAN models (see Fig. 13b).

Overall, by considering the complexity-vs.-training time-vs.-test time-vs.-accuracy tradeoff, we can argue that the proposed method can represent a promising approach for the robust classification of the COVID-19 disease from unlabeled CT scans.

## Conclusion and hints for future research

In this paper, we developed a KDE-based inference method, which leverages the hidden features extracted by BiGANs and CycleGANs for estimating, in the training phase, the (*a priori* unknown) PDF of the CT scans of COVID-19 patients (that is, the target COVID-PDF). Afterward, in the test phase, the distance (in the latent space) between the PDF of each test CT scan and the target COVID-PDF is evaluated, and, then, a tunable binary detector is implemented for generating the COVID/Non-COVID final decisions. We have numerically checked the implementation complexity-vs.-performance trade-offs attained by the designed BiGAN and CycleGAN models under several settings of training loss functions and distance metrics for test classification. In order to better corroborate the obtained numerical results, we have also checked the corresponding implementation complexity-vs.-performance trade-offs of some state-of-the-art competing models, which utilize the encoders of unsupervised-trained CAEs as feature extractors. The comparative analysis of the obtained numerical results supports the final conclusions that: i) the test accuracies of the proposed CycleGAN-based (resp., BiGAN-based) models outperform the corresponding ones of the benchmark CAE-based models of about 16% (resp., 14%); while, ii) the average training times of the tested CAE-based models are lower than the ones of the developed Cycle/BiGAN-based models of about 18–19 times.

The presented results open, indeed, the doors to five main research directions regarding the utilization of Cycle/Bi-GAN-based engines for image classification.

First, recovery of hyperspectral images (i.e., images composed by a number of inter-depending multispectral spatial slices) is an ill-posed (typically, nonconvex) constrained inverse problem, in which high-resolution multiband images must be recovered from their low-resolution (i.e., mixed and/or noise-affected) counterparts [67]. Recently, in [67, 68], supervised-trained CNN-based methods have been developed for unmixing and classification of hyperspectral images. Hence, developing effective Cycle/BiGAN-based models for the weakly supervised recovery/classification of hyperspectral images may be a first research topic of potential interest.

Second, in [68], supervised-trained graph convolutional networks (GCNs) (i.e., CNNs capable to operate on input data described by assigned adjacency graphs) have been designed for hyperspectral image classification. Motivated by the good performance reported in [68], we believe that an interesting research topic could concern the design of BiGAN and CycleGAN models that are capable to operate over graph-structured input data, in which long-range spatial dependence is captured by suitable adjacency matrices.

The recent contribution [69] proposes a CNN-based architecture for the joint extraction and fusion of features from multi-modal input data (i.e., heterogeneous input data that refer to a same object/scene to be classified). The design of novel BiGAN and CycleGAN architectures for multi-modal learning could be a third research line of potential interest.

A further hint for future research arises from the consideration that hyperspectral images are typically represented as data cubes with spatial-spectral information, in which non-negligible inter-data correlation is typically present along the spectral axis. To suitably exploit this correlation, the recent contribution in [70] proposes a new supervised-trained transformer-based DNN model (referred to as *SpectralFormer*) for the reliable classification of hyperspectral images. Hence, an interesting topic could concern the exploitation of BiGANs and CyCleGANs for the design of transformer-based DNN architectures that rely on weakly supervised training for image classification.

Finally, a potential drawback of the developed BiGAN and CycleGAN models is that their training times are quite long (i.e., more than 18 times larger than the corresponding ones of the tested CAE-based models). Hence, how to exploit Cloud/Fog-based [71, 72] virtualized [73] and (possibly) multi-antenna [74, 75] computing architectures for the parallel and distributed training of heavy BiGAN/CycleGAN models in interference-affected broadband wireless domains [76, 77] could be a final research topic of potential interest.

## Data Availability

The datasets generated during and/or analyzed during the current study are available in the COVID-Net repository (https://alexswong.github.io/COVID-Net/), and the Chest CT-Scan images Dataset (https://www.kaggle.com/datasets/mohamedhanyyy/chest-ctscan-images).
